# Incidence of Venous Thromboembolism in cancer patients treated with Cisplatin based chemotherapy — a cohort study

**DOI:** 10.1186/s12885-016-3032-4

**Published:** 2017-01-16

**Authors:** Muhammad Nauman Zahir, Quratulain Shaikh, Munira Shabbir-Moosajee, Adnan Abdul Jabbar

**Affiliations:** 1 Department of Oncology, Aga Khan University Hospital, Stadium Road, PO BOX: 3500, Karachi, 74800 Pakistan; 2 Department of Medicine, Aga Khan University Hospital, Stadium Road, PO BOX: 3500, Karachi, 74800 Pakistan

**Keywords:** Cisplatin, Chemotherapy, Venous thromboembolism

## Abstract

**Background:**

Cancer related thrombosis not only increases morbidity and mortality but also poses a significant financial burden on health care system. Risk of venous thromboembolism (VTE) in these patients substantially increases with the addition of chemotherapy. Lately, cisplatin has been implicated as an independent factor. There is little data estimating the risk of venous thromboembolism in patients receiving cisplatin based chemotherapy when compared to other chemotherapeutic agents.

**Methods:**

Patients who had received chemotherapy between November 2010 and October 2012 were retrospectively identified from a single institute cancer registry. 200 patients who had received cisplatin based chemotherapy were identified as the exposed group while 200 patients who had received non-Cisplatin based regimens were identified as the non-exposed group. Patients were followed for development of VTE throughout the entire duration of therapy and one month thereafter. Cox proportional hazard model was used to compute relative risks with 95% confidence intervals.

**Results:**

The baseline characteristics were similar in the two groups. Mean age for the entire cohort was 55.4 ± 10.7 years and male to female ratio was almost 1:1. On univariate analysis, cisplatin based chemotherapy, presence of central venous catheter, female gender, poor performance status, high risk stratification according to the Khorana model and use of granulocyte colony stimulating factor were all significantly associated with the development of VTE. The crude relative risk for the incidence of VTE in cisplatin group was 2.8 (95% CI, 1.4 – 4.2) times compared to the non-Cisplatin group. When the relative risk was adjusted for the above variables in multivariable analysis, it increased to 3.3 (95% CI, 1.6 – 6.8) compared to the control group.

**Conclusion:**

A high incidence of VTE in patients receiving cisplatin based chemotherapy was demonstrated in this study. Prospective studies are warranted to establish this observation with certainty and to explore the possible use of thromboprophylaxis in patients receiving cisplatin based chemotherapeutic regimens.

## Background

There is a substantial risk of venous thromboembolic events (VTE) in patients with cancer. The incidence of VTE in the general population is approximately 117 per 100,000, whereas the incidence in patients with cancer is approximately one in 200 [1, 2]. A large cohort study showed that the baseline risk of thrombosis in cancer is approximately 4.1 folds compared to the healthy population while it increases to 6.5 fold after the addition of chemotherapy [3]. This leads to reduction in survival of patients with malignancy and increased expense of treatment and care [4–6]. Although individual factors including the tumor type, stage, recency of cancer diagnosis, presence and number of co-morbidities define risk of VTE in cancer, active therapy with certain chemotherapeutic agents has been contemplated as an additional risk factor [7]. There is anecdotal evidence of increased incidence of VTE in patients receiving Cisplatin based regimes [8, 9]. Studies in urothelial, germ cell and non-small cell lung cancer (NSCLC) patients have established a link between Cisplatin and increased incidence of VTE [10–12]. Another recent large retrospective study showed the prevalence of VTE to be 16.6% in all patients treated with Cisplatin [13] which is significantly higher than 7.3% for VTE in patients treated with any chemotherapy [14]. Prospective randomized controlled trials in patients with advanced esophagogastric junction (EGJ) or gastric adenocarcinoma showed that this thrombogenic effect is not class specific for all the platinum containing chemotherapeutic agents, but only Cisplatin based chemotherapy is associated with a statistically significant greater number of VTE events than Oxaliplatin [15, 16]. Recently, a meta-analysis from case series and some pre-clinical trials provided evidence of high risk of VTE in patients receiving Cisplatin based regimens [17].

Various hypotheses exist pertaining to the mechanisms associated with Cisplatin associated thrombosis including direct damage to the vascular endothelium, increased procoagulant activity, reduced anticoagulation synthesis, platelet activation and aggregation and vascular inflammation [18–24].

Despite increasing evidence pertaining to the thrombogenic potential of Cisplatin, no original analytical study has thus far investigated the magnitude of increased risk of VTE imparted by Cisplatin based chemotherapies when compared to other chemotherapies. Hence, it is extremely important to quantify this risk in order to advocate prophylaxis with antiplatelets or anticoagulants and hence reduce the economic and health care associated burden of VTE caused by Cisplatin based chemotherapy. Therefore we aimed to quantify the risk and identify high risk groups so that treatment is targeted and benefits maximized. Prophylactic anticoagulation for all cancer patients is expensive and has a narrow therapeutic index and hence not practical. We hypothesized that there is an increased risk of VTE in cancer patients receiving Cisplatin based chemotherapy regimens compared to other chemotherapies.

## Methods

This is a retrospective cohort study including all adult cancer patients presenting to the oncology ward or day care centre of the Aga Khan University Hospital for chemotherapy. Cancer patients receiving Cisplatin based chemotherapy regimens were considered exposed while those cancer patients receiving chemotherapy regimens which do not contain Cisplatin comprised the unexposed group. Adult (>16 years) participants were considered eligible if they received treatment between November 1, 2010 and October 31, 2012. They should have initiated and completed all treatment at Aga Khan University Hospital while maintaining a followup of atleast 1 month post completion of treatment or until death, whichever came first. Excluded were those on antiplatelets agents or anticoagulants, with a previous history of DVT or PE (self-reported), known pro-thrombotic disorders or hyper-coagulable states, patients who were on erythropoiesis-stimulating agent (ESA) during chemotherapy or up to 6 weeks prior to the first cycle of chemotherapy and those female patients who were on oral contraceptive pills.

**Fig. 1:**
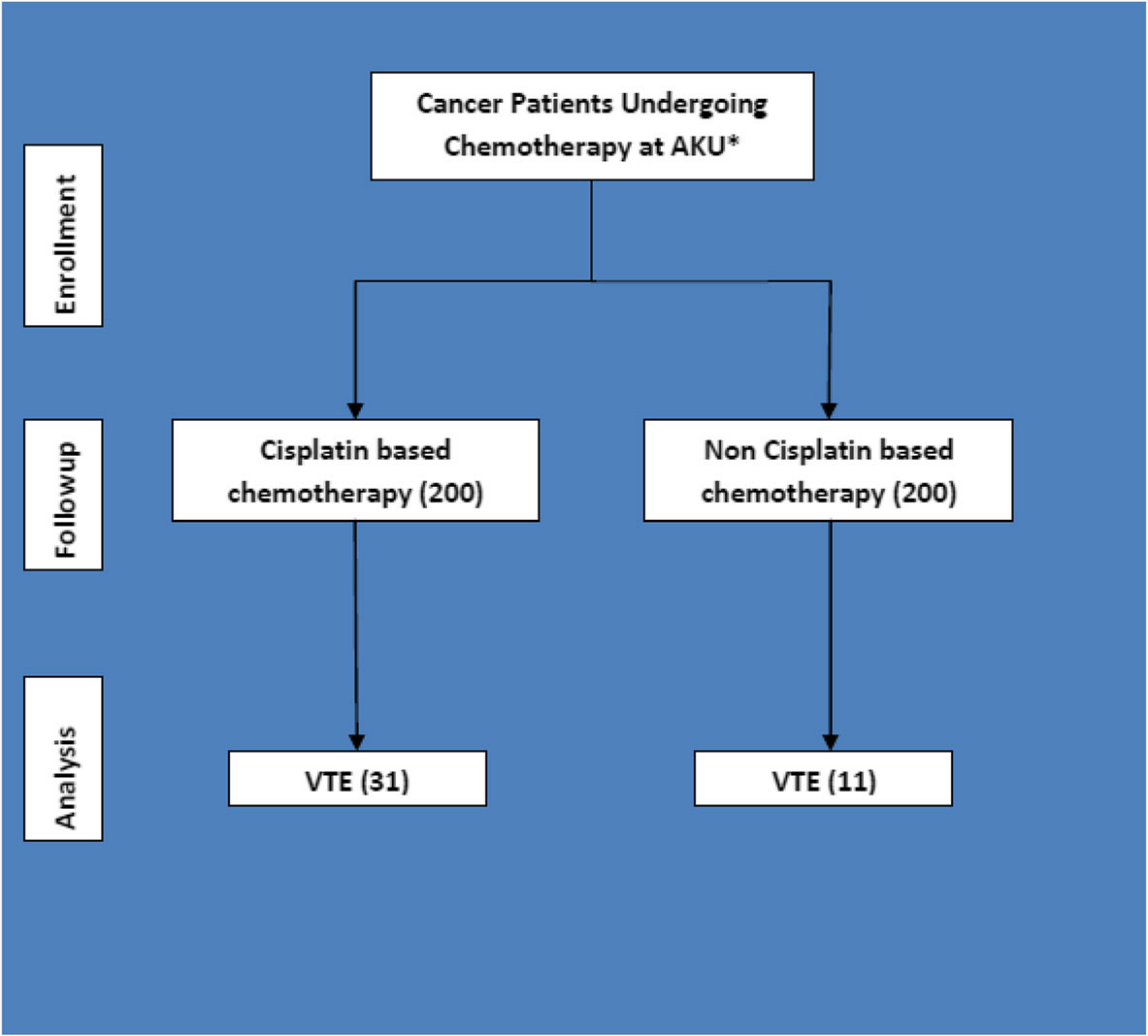
Flow chart of participants in the study. *Aga Khan University Hospital, Karachi, Pakistan

All cancer patients treated in the day care centre or the inpatient oncology wards of Aga Khan University Hospital between November 1, 2010 and October 31, 2012 were identified through the discharge coding system. 100 consecutive eligible patients were included in each group starting from November 1, 2010 (Fig. 1). Medical record files were reviewed for demographic and clinical data which was recorded on a pre-designed questionnaire. Confounding factors such as the presence of indwelling central line, obesity and family history of DVT were recorded if present. All identified patients were followed up during the course of chemotherapy and at least 4 weeks post chemotherapy. Radiological investigations for the study population were also reviewed to make sure that no cases of VTE are missed. In case of discrepancy between the chart data and radiological investigations, the patient was telephonically evaluated for the history of VTE event or the use of anticoagulant agents during the specified time period. Outcome was any VTE event during chemotherapy or within 4 weeks after the completion of chemotherapy. ECOG classification was modified for analysis as class 0, 1 and 2 were combined as “good”, while class 3 and 4 was “poor”. Class 5 was excluded. Khorana score was categorized as low risk (0 points), intermediate risk (1–2 points) and high risk (3 or more points).

## Outcome

### Venous thromboembolism

Venous Thromboembolism was defined as the occurrence of a thromboembolic event after atleast 3 days of initiation of chemotherapy up to 4 weeks of the completion of chemotherapy cycle. This included any deep venous thrombosis and/or pulmonary embolism as under:

1. Evidence of venous thromboembolism on Computed Tomography Chest.
2. Evidence of DVT anywhere in the body on radiological evaluation including ultrasound, CT scan or MRI within the period defined above
3. Cause of death/morbidity defined as Pulmonary Embolism by primary physician.
4. For patients who develop signs and symptoms of VTE but died before diagnostic criteria could be met, sudden, otherwise unexplained death was taken as a VTE event in the presence of severe hypoxia on ABGs and no acute change in chest X-ray.

With a confidence level of 95%, power of 80% and considering the prevalence of venous thromboembolism in cancer patients receiving chemotherapy to be around 7.3% while the anticipated prevalence after receiving Cisplatin chemotherapy to be 16.6% the minimum sample size in each group to refute the hypothesis of equality of proportions was 200 [14]. Patients were enrolled after informed consent. Ethical approval was taken from the Institutions’ Ethics Committee under approval number 2703-Med-ERC-2013. Complete confidentiality of all patient information and personal data was ensured.

### Statistical analysis

Mean ± SD was reported for continuous variables and percentages and proportions for categorical variables. Cox proportional hazard model was used to calculate crude and adjusted Relative risks and 95% confidence interval for incidence of VTE. *p* value < 0.05 was considered significant. Data was analysed using Stata version 12.

## Results

The mean age of Cisplatin group was 56(11.9) years while in the non-Cisplatin groups it was 55(9) years. There was a slight preponderance of males in both groups (Table 1). Most of the cancers were metastatic in both groups and very few were early stage malignancies (Tables 2 and 3). Less than 10% of the whole cohort had ECOG poor status. 129(64.5%) participants in Cisplatin group had intermediate Khorana risk score as compared to 125(62.5%) in the Non-Cisplatin group. 29% of the patients had an indwelling central venous catheter (CVC) at some point during the study in the Cisplatin group vs. 27% in the non-Cisplatin group. Details of the chemotherapy regimens used in both groups have been highlighted in Tables 4 and 5 whereas important co-morbids in the two cohorts have been summarized in Table 6.

**Table 1:** Baseline Characteristics of the participants

Variables	Cisplatin Group *n* = 200 *n* (%)	Non-Cisplatin Group *n* = 200 *n* (%)
1 Age (yrs)^a^	56 (11.9)	55 (9)
2 Males	108 (54%)	104 (52%)
3 Time to VTE (days)^a^	58 (17)	56 (13)
4 Stage		
Early	10 (5%)	12 (6%)
Locally advanced	86 (43%)	92 (46%)
Metastatic	104 (52%)	96 (48%)
5 Presence of CVC	58 (29%)	55 (27%)
6 Surgery in 2 months	44 (22%)	56 (28%)
7 ECOG poor	16 (8%)	12 (6%)
8 Khorana Risk		
low	39(19.5%)	55 (27.5%)
intermediate	129 (64.5%)	125 (62.5%)
high	32 (16%)	20 (10%)
9 Use of GCSF		
never	88 (44%)	86 (43%)
<50% of cycles	71 (35.5%)	81 (40.5%)
>50% of cycles	6 (3%)	24 (12%)
All cycles	35 (17.5%)	(9 9.5%)

^a^Mean(SD)

**Table 2:** Cancer type and stage of patients receiving cisplatin based chemotherapy

Site of cancer	Stage of tumor (*N* = 200)			Total (%)
	Early (%)	Locally advanced (%)	Metastatic (%)	
Lung	1 (0.5)	16 (8)	21 (10.5)	38 (19)
Gastric/GEJ	2 (1)	8 (4)	10 (5)	20 (10)
Head and Neck	1 (0.5)	14 (7)	19 (9.5)	34 (17)
Pancreatic	0	4 (2)	9 (4.5)	13 (6.5)
Ovarian	1 (0.5)	5 (2.5)	0	6 (3)
Esophageal	1 (0.5)	6 (3)	9 (4.5)	16 (8)
Germ Cell	3 (1.5)	1 (0.5)	9 (4.5)	13 (6.5)
Cervical/Vulvar	0	3 (1.5)	7 (3.5)	10 (5)
Bladder	0	5 (2.5)	7 (3.5)	12 (6)
Endometrial	1 (0.5)	3 (1.5)	0	4 (2)
Chloangiocarcinoma	0	2 (1)	2 (1)	4 (2)
Breast	0	3 (1.5)	3 (1.5)	6 (3)
Carcinoma of unknown primary (CUP)	0	6 (3)	2 (1)	8 (4)
Gall bladder/peri-ampullary	0	4 (2)	3 (1.5)	7 (3.5)
Lymphoma^a^	0	9 (4.5)		9 (4.5)

^a^Lymphoma can only be classified into early or advanced stage

**Table 3:** Cancer type and stage of patients receiving non-cisplatin based chemotherapy

Site of cancer	Stage of tumor (*N* = 200)			Total (%)
	Early (%)	Locally advanced (%)	Metastatic (%)	
Lung	1 (0.5)	6 (3)	7 (3.5)	14 (7)
Gastric/GEJ	0	8 (4)	8 (4)	16 (8)
Pancreatic	0	8 (4)	6 (3)	14 (7)
Ovarian	0	7 (3.5)	9 (4.5)	16 (8)
Endometrial	0	3 (1.5)	3 (1.5)	6 (3)
Breast	4 (2)	28 (14)	28 (14)	60 (30)
Colorectal/Anal/Small bowel	5 (2.5)	17 (8.5)	22 (11)	44 (22)
Lymphoma^a^	1 (0.5)	11 (5.5)		12 (6)
Sarcoma	1 (0.5)	7 (3.5)	4 (2)	12 (6)
Others	0	1 (0.5)	5 (2.5)	6 (3)

^a^Lymphoma can only be classified into early or advanced stage

**Table 4:** Chemotherapy regimens received by patients in the cisplatin group

Type of chemotherapy	Number of patients (%) *N* = 200
Cisplatin + Gemcitabine	58 (29)
Cisplatin + Concurrent chemoradiation	28 (14)
Cisplatin + 5-FU/Cisplatin + Capecitabine	8 (4)
Cisplatin + 5-FU + Radiation/Cisplatin + Capecitabine + Radiation	7 (3.5)
Docetaxel + Cispaltin + 5-FU	30 (15)
Epirubicin + Cisplatin + 5-FU/Epirubicin + Cisplatin	10 (5)
Docetaxel + Cispaltin + 5-FU + Cetuximab	4 (2)
DHAP (Dexamethasone + High dose ARA-C + Cisplatin)	8 (4)
Cisplatin + Etoposide/Cisplatin + Etoposide + Bleomycin	12 (6)
Cisplatin + Pemetrexed	16 (8)
Cisplatin + Navelbine	15 (7.5)
Cispaltin + Gemcitabine + Radiation	4 (2)

**Table 5:** Chemotherapy regimens received by patients in the non-cisplatin group

Type of chemotherapy	Number of patients (%) *N* = 200
Capecitabine + Oxaliplatin/5-FU + Leucovorin + Oxaliplatin/Epirubicin + Oxaliplatin + Capecitabine	34 (17)
5-FU + Irinotecan + Leucovorin/5-FU + Irinotecan + Leucovorin + Cetuximab/Irinotecan + Cetuximab	18 (9)
Doxorubicin + Cyclophophamide/5-FU + Epirubicin + Cyclophophamide/Epirubicin + Cyclophophamide	24 (12)
Paclitaxel/Paclitaxel + Herceptin	22 (11)
Docetaxel/Docetaxel + Herceptin	26 (13)
Adrimacycin + Ifosfamide	8 (4)
Carboplatin + Paclitaxel	34 (17)
CHOP/ABVD (Cyclophosphamide + Adriamycin + Vincristine + Prednisone)/(Adriamycin + Bleomycin + Vinblastine + Dacarbazine)	12 (6)
VAC alternating with IE (Vincristine + Adriamycin + Cyclophosphamide alternating with Ifosfamide + Etoposide)	4 (2)
5-FU + Oxaliplatin + Irinotecan + Leucovorin	8 (4)
Gemcitabine	6 (3)
Carboplatin + Gemcitabine	2 (1)
Others	2 (1)

**Table 6:** Comparison of the co-morbidities present in the two groups

Co-morbid	Number in cisplatin group (%)	Number in noncisplatin group (%)
	*N* = 200	*N* = 200
Diabetes Mellitus	30 (15)	24 (12)
Hypertension	46 (23)	52 (26)
Coronary artery disease	10 (5)	08 (4)
Prior Atrial fibrillation	04 (2)	04 (2)
Prior history of TIA/Stroke	02 (1)	00
Renal Failure	00	00
Heart Failure	00	00

When the dose of Cisplatin was considered, we found that 31 VTE events occurred when the mean (SD) cumulative dose of Cisplatin was 471(133) mg/m^2^ while in the group without events the mean cumulative dose was 322(124) mg/m^2^ (Table 7).

**Table 7:** Effect of Cisplatin dose on incidence of VTE

VTE status	Cumulative dose of Cisplatin (mg/m^2^) Mean (SD)
VTE occurred *n* = 31	471 (133.4)
VTE did not occur *n* = 169	322 (124)

Among the VTE events most were DVTs i.e.18 in Cisplatin group while 7 in non-Cisplatin group (Appendix 1). Among those who suffered a VTE event, 4 died in Cisplatin group while 1 in non-Cisplatin group (Appendix 2). On univariate analysis other than Cisplatin based chemotherapy, poor ECOG status, presence of CVC and Khorana risk score were statistically significant (Appendix 3). These were used for final model building.

The crude relative risk of VTE in the Cisplatin group was 2.8 (95% CI: 1.4–4.22) times higher than in the Non-Cisplatin group (Table 8). When adjusted by gender, ECOG status, GCSF, presence of CVC and Khorana risk score the adjusted relative risk was 3.32(95% CI:1.6–6.8) (Table 9).

**Table 8:** Crude Relative Risk of Venous Thromboembolism in Cisplatin based regimens

	Incidence proportion	Relative Risk (95% CI)
Cisplatin group	31/200	2.81* (1.4–4.2)
Non-Cisplatin group	11/200	

*p < 0.05

**Table 9:** Adjusted Relative Risk of VTE due to Cisplatin based Chemotherapy

	β coefficient	SE (β)	Relative Risk	95% CI for RR
Cisplatin based chemotherapy	1.2	0.37	3.32	1.6–6.8

adjusted for ECOG, Khorana risk score, GCSF, presence of CVC and gender-log likelihood =471.38

## Discussion

This study reports a high risk (RR: 3.3, 95% CI: 1.6–6.8) of VTE in cancer patients receiving Cisplatin based chemotherapy. Cisplatin is a platinum based drug which is used for the treatment of cancer. The mechanism of causing VTE is not clear but endothelial injury, hypomagnesemia and increased levels of Von Willebrand’s factor are involved [8, 19–21, 23]. Patient factors including female sex, age, type of cancer, Khorana risk and indwelling CVC further elevate the risk of VTE [13]. In our study Khorana risk and presence of CVC were found to be significantly associated with this risk. Another interesting finding, which has not been explored previously, is the risk of VTE with higher cumulative doses of Cisplatin (Table 3). In our study, cumulative Cisplatin dose of more than 450 mg was associated with VTE events.

Our risk estimates are higher than a previously reported meta-analysis where the risk was 1.67 (95% CI: 1.25–2.23). The reason for this may be Seng et al, in their meta-analysis pooled results from phase II to III clinical trials. It is possible that patients in their study were a highly selective group. Another explanation could that we had highly specialised investigations available and the chances of diagnosing a VTE are higher in the present era than between 1990 and 2010 which is the study time for Seng et al’s study [17].

The reason for choosing one month follow-up was twofold. One, previous literature [13] had also taken one month follow-up for development of DVT in patients receiving Cisplatin based chemotherapy. This study reported that 88% of patients developed TEE within 100 days of initiation of treatment with Cisplatin based regimens. Hence a period of 30 days post completion of chemotherapy seems a reasonable follow-up period for capturing most, if not all, events occurring as a result of the drug intervention. Secondly, we believe it is safe to assume that since re-dosing of Cisplatin as a chemotherapeutic agent is required within 3–4 weeks of the last dose; most therapeutic as well as adverse effects of the drug are neutralised within one month of last dosing. Moreover, if a VTE occurs one month after completion of chemotherapy, it becomes further complex to attribute it solely to chemotherapy effects as underlying disease and other risk factors (e.g. CVC, immobility etc.) are likely to play a much more important role.

Our major strength is being the first original research reporting relative risk of VTE in Cisplatin based regimens compared to non-Cisplatin. Aga Khan University hospital is one of the two large centres for the treatment of cancer in Pakistan. Hence, we were able to obtain a reasonable sample with reliable data on patients from the time of starting Cisplatin therapy up till one month post chemotherapy. We were able to maintain close follow up with all our patients without attrition. There was no missing information because we had access to medical records and patients were coming for clinical follow ups regularly. We chose a retrospective study design for feasibility of sample size achievement and save resources.

The data was collected retrospectively but since all the parameters were measured and documented in hospital notes we assume there is minimal bias due to recall. Although we may have missed some important confounders, one advantage of using hospital data in this study was that the medical details were very accurate and radiological investigations were easy to follow hence minimizing misclassification of exposure and outcome.

Site of cancer could be an important confounder hence it was incorporated as one of the predictors of Khorana score and it is reflected within the scoring. Accordingly, we have adjusted our final model for Khorana score to eliminate any confounding effect that may have arisen due to the site of disease.

Polychemotherapy may potentially attribute higher rates of VTE than single agent chemotherapy but this fact is unlikely to affect our results as almost all patients in both the groups received combination chemotherapy (Tables 4 and 5). Patients who received other platinum based therapies (Carboplatin, Oxaliplatin), were included in the non-Cisplatin group further highlighting the possibility that the increased thrombogenic effect may be inherent to Cisplatin alone rather than to the entire group of platinum drugs.

This however, is also a potential bias and limitation of our study as a difference in VTE incidence between the different platinum drugs was not explored further. Our study had not been designed to evaluate for any differences in VTE incidence between the platinum compounds but further dissection and analysis could indeed prove very interesting and is an area which needs to be explored in future studies.

Another potential limitation of our study is the absence of a universal screening test for assessment of VTE status prior to initiation of chemotherapy in patients. Although this would have been the ideal method to ensure absence of VTE (target end-point) in newly recruited patients, the retrospective nature of our study meant that we had no control over investigations performed both prior to and during the course of the study.

In the light of these finding we think there is an urgent need to prospectively estimate the risk of VTE in patients receiving Cisplatin based regimens. It is immensely important to discover the pathophysiology and identify genetic and molecular mechanisms of occurrence of VTE in these patients. Till then we strongly recommend prophylaxis against VTE in high risk groups to avoid serious morbidity or mortality. We emphasize that it is extremely important to develop potent and effective chemotherapeutic agents with fewer side effects.

## Conclusion

In our experience this is one of the first reports of risk estimates of VTE with Cisplatin based chemotherapeutic regimens. Focus should be laid on discovery of newer and safer chemotherapeutic agents. Till then, prophylaxis against VTE for patients receiving Cisplatin based chemotherapy is highly recommended. It is important to identify high risk groups vulnerable to this side effect.

## Appendix 1

**Table 10:** Venous thromboembolic Events in Cisplatin based and Non-Cisplatin based Chemotherapeutic regimes

Venous Thromboembolic Events	Cisplatin group	Non-Cisplatin group	*p* value
	*n* = 200	*n* = 200	
DVT alone	18	7	
PE alone	7	2	
DVT + PE	5	2	
Sudden death (defined as VTE)	1	0	
Total	31	11	0.02

## Appendix 2

**Table 11:** Patient outcomes after Venous Thromboembolic Events

Patient status after VTE	Cisplatin group *N* = 31	Non-Cisplatin group *N* = 11
Dead	4	1
Alive	27	10

## Appendix 3

**Table 12:** Risk factors for venous thromboembolism in cancer patients

Variable	β coefficient	SE	*P* value
1 Cisplatin based chemotherapy	1.034	0.35	0.003
2 Age (years)	0.01	0.015	0.42
3 ECOG poor	0.79	0.44	0.07
4 Female	0.41	0.31	0.19
5 GCSF never	0		
< 50% of the cycles	0.13	0.37	
> 50% of the cycles	0.87	0.45	
all	0.15	0.51	0.17
6 Stage early	0		
locally advanced	−0.42	0.63	
metastatic	−0.17	0.61	0.67
7 CVC present	0.74	0.31	0.01
8 Khorana Risk low	0		0.07
intermediate	−0.91	0.46	
high	−0.85	0.37	

## Abbreviations

ABG: Arterial blood gases
CT: Computed tomography
CVC: Central venous catheter
DVT: Deep venous thrombosis
ECOG: Eastern cooperative oncology group
EGJ: Esophagogastric junction
ESA: Erythropoiesis stimulating agent
MRI: Magnetic resonance imaging
NSLC: Non-small cell lung cancer
SD: Standard deviation
VTE: Venous thromboembolism
